# A Retrospective Clinicopathological Study of 37 Patients With Chordoma: A Danish National Series

**DOI:** 10.1080/13577149778254

**Published:** 1997-12

**Authors:** Akmal Safwat, Ole S. Nielsen, Anne G. Jurik, Johnny Keller, Ernst R. Weeth, Bjarne Lund, Olaf Myhre-Jensen

**Affiliations:** 1 Sarcoma Centre of Aarhus University Hospital Aarhus C DK-8000 Denmark; 2 Sarcoma Centre of Odense University Hospital Denmark; 3 Rigshospitalet Denmark; 4 Department of Oncology Aarhus University Hospital Norrebrogade 44 Aarhus C DK-8000 Denmark

## Abstract

*Purpose*. There are, in general, few published series on chordoma. It is a rare disease and further data are still needed.

*Patients/methods*. The data of 37 patients with chordoma were retrospectively analyzed. Treatment was surgical excision in 11, radical radiotherapy in 9 and a combination of the two in 16 cases. The male to female ratio was 2.7 : 1. Median age was 59 years (range 1–89 years).

*Results*. The most common symptoms at diagnosis were pain (98%), neurological disturbances (42%) and incontinence (33%). The tumours were located in the sacro-coccygeal region in 68%, the spheno-occipital region in 16% and the vertebrae in 16% of the patients. Median tumour.size was 7 cm (range 1–30 cm). Local recurrence occurred in 21/36 treated cases and distant metastases developed in eight patients (23%). The median time to recurrence/progression after primary treatment was 2 years (range 1–10 years). The actuarial 5-year rates of overall, progression-free and symptom-free survival were 40%, 31% and 20%, respectively. The corresponding 10-year rates were 26%, 21% and 14%, respectively. At the time of analysis, seven patients were alive, six without evidence of disease. Four of the six patients without active disease were symptom free. A univariate analysis showed that age, sex, tumour size, histopathology, surgical safety margin, treatment modality and radiation dose did not significantly affect overall, progression-free or symptom-free survival. Only turnout site had a prognostic value with turnouts in the spheno-occipital region carrying the worst prognosis.

*Discussion*. We conclude that effective treatment against chordomas is still lacking and a prospective multi-institutional registration study may provide more information on the optimal work-up and treatment of this rare disease.


*Sarcoma* (1997) 1, 161–165

CARFAX

ORIGINAL ARTICLE

### A retrospective clinicopathological study of 37 patients with chordoma: a Danish national series

AKMAL SAFWAT,[1] OLE S. NIELSEN,[1] ANNE G. JURIK,[1] JOHNNY KELLER,[1] ERNST R. WEETH,[2] BJARNE LUND[3] & OLAF MYHRE-JENSEN[1]

*Sarcoma Centres of [1]Aarhus University Hospital, [2]Odense University Hospital & [3]Rigshospitalet, Denmark*

**Abstract**

*Purpose.* There are, in general, few published series on chordoma. It is a rare disease and further data are still needed. *Patients/methods.* The data of 37 patients with chordoma were retrospectively analyzed. Treatment was surgical excision in 11, radical radiotherapy in 9 and a combination of the two in 16 cases. The male to female ratio was 2.7:1. Median age was 59 years (range 1–89 years).

*Results.* The most common symptoms at diagnosis were pain (98%), neurological disturbances (42%) and incontinence (33%). The tumours were located in the sacro-coccygeal region in 68%, the spheno-occipital region in 16% and the vertebrae in 16% of the patients. Median tumour size was 7 cm (range 1–30 cm). Local recurrence occurred in 21/36 treated cases and distant metastases developed in eight patients (23%). The median time to recurrence/progression after primary treatment was 2 years (range 1–10 years). The actuarial 5-year rates of overall, progression-free and symptom-free survival were 40%, 31% and 20%, respectively. The corresponding 10-year rates were 26%, 21% and 14%, respectively. At the time of analysis, seven patients were alive, six without evidence of disease. Four of the six patients without active disease were symptom free. A univariate analysis showed that age, sex, tumour size, histopathology, surgical safety margin, treatment modality and radiation dose did not significantly affect overall, progression-free or symptom-free survival. Only tumour site had a prognostic value with tumours in the spheno-occipital region carrying the worst prognosis.

*Discussion.* We conclude that effective treatment against chordomas is still lacking and a prospective multi-institutional registration study may provide more information on the optimal work-up and treatment of this rare disease.

**Key words:** *chordoma, treatment results, survival, univariate analysis.*

### Introduction

Chordomas are rare tumours that arise along the axial skeleton with the sacro-coccygeal and the spheno-occipital regions being the two most favoured sites.[1,2] They are generally considered to arise from remnants of notochordal tissue.[3] Surgery and radiation therapy are the main lines of treatment. However, the close proximity of these tumours to critical neural and skeletal tissues can compromise both the radical approach of surgical resection and radiation dose. Radiotherapy is usually recommended in inoperable cases, following surgery, or for the treatment of gross recurrence.[4] The timing of radiotherapy and the optimum radiation dose and fractionation have not been established.[5] Local control is poor and death is most often caused by consequences of uncontrolled local tumour growth.[6] Distant metastases are not uncommon but their frequency varies widely between published series.[1,7] No chemotherapeutic regimen has been shown to be effective against these tumours.[4,8]

In general, there are few published series on chordoma, and because of the rarity of the disease few publications contain more than 25 patients. No prospective randomized studies have been reported. Hence, in most cases, the available data in the literature represent selected patients. This may explain partially the discrepancies between results, and more clinical reports are therefore still needed. This paper represents the first published description of the Danish experience in the management of patients with chordoma.

### Patients and methods

This national Danish study of 37 patients represents all the patients treated in the three major sarcoma centres in Denmark in the last 25 years. The files of all patients were reviewed and all histopathological

Correspondence to: O. S. Nielsen, Department of Oncology, Aarhus University Hospital, Norrebrogade 44, DK-8000 Aarhus C, Denmark. Fax: + 45 89492550; E-mail: osn@onko.aau.dk.

1357-714X/97/030161-05 $9.00 © 1997 Carfax Publishing Ltd specimens were reviewed centrally by one of the authors (OM-J).

Primary treatment was surgical resection in 11 patients, radical radiotherapy in 9 patients and combined surgical excision and post-operative radiotherapy in 16 patients. One patient did not receive any treatment after tumour biopsy. It was not clear in the file why this was so. The major goal of surgery was total tumour removal, but such surgery was only considered feasible in four patients with chordomas in the sacro-coccygeal region. Over this long study period the operative approaches varied according to both the location of the tumour and the experience of the surgeons. Chemotherapy was not given as part of the primary treatment. One patient was lost to follow-up. The median time of follow-up was 62 months (range 4–236 months).

The therapeutic results were evaluated in terms of symptomatic relief, local control and survival. Overall survival, progression-free survival and symptom-free survival were calculated using the actuarial approach,[9] and differences were tested by the log-rank test.[10] The log-rank test was used as a univariate analysis of actuarial estimates in an attempt to elucidate the role of various possible prognostic factors. These factors included: age, sex, tumour size, tumour site, surgical safety margin, treatment modality and radiation dose.

### Results

### *Natural history*

In Denmark, between one and five chordomas are diagnosed per year. In the present series, there were 27 males and 10 females, i.e. the male to female ratio was 2.7:1. The age of patients ranged from 1 to 89 years with a median age of 59 years. Median duration of symptoms before admission was 12 months (range 1–84 months). Symptoms dominating at diagnosis were pain (98%), neurological disturbances (42%) and incontinence (33%).

The tumours were located in the sacro-coccygeal region in 25 patients (68%), spheno-occipital region in six patients (16%) and vertebrae in six patients (16%). The median value of 'maximum tumour diameter' was estimated to be 7 cm (range 1–30 cm), with the large tumours mainly seen at the sacral region; however, it should be noted that the methods used to measure tumour size varied from simple clinical judgement to measurements based on magnetic resonance scanning.

### *Treatment results*

Of the 27 patients in whom an attempt at surgical excision was part of their initial treatment, a wide safety margin could be achieved in only four cases (all sacral). Surgery was intra-lesional in 17 cases and marginal in six cases. After the initial primary surgery, only the four patients with wide safety margins were evaluated as being in complete remission.

Radiotherapy was given with conventional fractionation (1.8–2.5 Gy/fraction, 5 fractions/week) in 15 patients. High dose per fraction (3–4 Gy/fraction) was used in four patients and multiple fractions per day with small dose per fraction (1–1.7 Gy/fraction) in five patients. In one patient the fractionation schedule was not reported. The median total radiation dose was 55 Gy (range 30–80 Gy) and the median number of fractions 29 (range 13–50 fractions). In the group treated with radiation alone the median dose was 57 Gy and the median number of fractions 24, and in the group treated with combined surgery and radiation the median dose was 55 Gy and the median number of fractions 30.

The time to recurrence (local or distant) following primary treatment ranged from 1 to 10 years with a median of 2 years. The one patient who did not receive any primary treatment after biopsy had progression of his local disease 14 years after diagnosis. This was eventually the cause of his death. Local recurrence was defined as pathological or radiological evidence of disease progression occurring more than 6 months after treatment. Local recurrence occurred in 21/36 treated patients (58%). Distant metastases developed in 8/36 treated patients (23%). In 5/8 cases, metastases were not accompanied by local disease.

Treatment of local recurrence was surgical in four cases, radiotherapy in three cases and surgery plus post-operative radiotherapy in six cases. In eight cases, no treatment was given, mainly because of poor general condition or inoperability. Chemotherapy was given to 3/8 metastatic cases, and the regimens used were single-drug doxorubicin, single-drug ifosfamide and CYVADIC (cyclophosphamide, vincristine, doxorubicin, dacarbazine), respectively. The time from recurrence to death ranged from 0 to 84 months with a median of 15 months. However, the median time from the diagnosis of progression to death was 26.5 months (range 5–84 months) for the treated group and 14.5 months (range 4–28 months) for the untreated group.

At the time of analysis, seven patients were alive, six without active disease. Four of the six patients alive without active disease were symptom free. Progression of disease was the cause of death in 18 patients. In three patients with active disease, death was due to other, unrelated, diseases. In three patients with active disease, death was directly attributed to treatment complications. One patient with a chordoma in the spheno-occipital region died 8 days after surgery probably due to cerebral oedema. one died of an osteosarcoma probably secondary to the irradiation, and one died of gastrointestinal complications probably related to the irradiation. In four patients the cause of death was not defined and only one patient died from an unrelated cause while in complete remission.

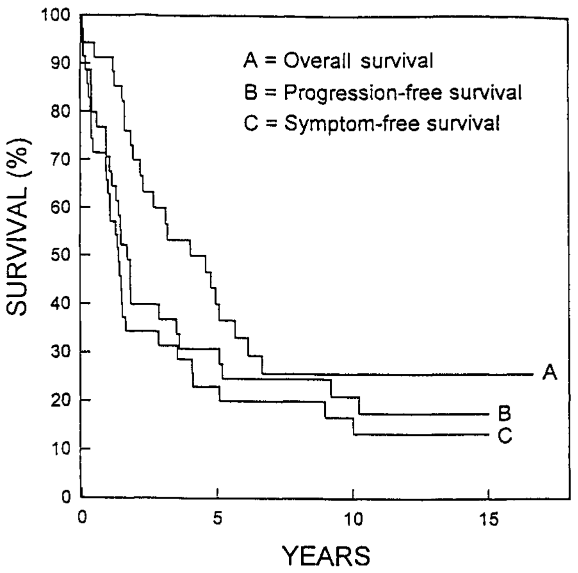

**Fig. 1.** *Acturial (Kaplan–Meier) estimate of overall survival, progression-free survival and symptom-free survival for all patients over an extended period of follow-up.*

The actuarial 5-year rates of overall, progression-free and symptom-free survival (± 1 SE) were 40% (± 9%), 31% (± 8%) and 20% (± 7%), respectively. The corresponding 10-year rates were 26% (± 8%), 21% (± 7%) and 14% (± 6%) for overall, progression-free and symptom-free survival, respectively (Fig. 1).

*Prognostic factors*

As seen in Table 1, the outcome of therapy (5-year overall, progression-free and symptom-free survival) did not differ between the various treatment groups. Table 2 shows that with the exception of tumour site, none of the prognostic factors studied was found to affect survival, progression-free survival or symptom-free survival. Continuous variables (age, tumour size and radiation dose) were divided into two at the median values. To compensate for the variation in fractionation schedules the total dose was calculated in terms of biological effective dose (BED) using the equation:

$$BED = nd\ (1 + d/\alpha/\beta)$$

where $n$ = number of fractions, $d$ = dose per fraction and $\alpha/\beta$ for chordoma was estimated to be 7 Gy. Still, no effect of BED was found on survival, progression-free survival and symptom-free survival.

Patients with chordomas of the spheno-occipital region did much worse than patients with chordomas in any other site. None of the patients with spheno-occipital tumours survived for 5 years. This difference in survival was statistically significant ($p = 0.001$), while there was no difference in survival between vertebral and sacral chordomas (Fig. 2).

### Discussion

Because of the rarity of the disease, this series of 37 patients is actually one of the largest in the published literature. The natural history and clinicopathological criteria of the chordoma reported in this study agree with most of the reported figures. These include the median age of 59 years[1,11,12] and a male to female ratio of around 2 : 1[7,12] Chordomas are known to have a slow rate of progression. The duration of symptoms before diagnosis is therefore relatively long[1,11] and agrees perfectly with the median duration of 12 months reported here. Spheno-occipital chordomas represented 16% of our cases. This value is lower than the 35–68% incidence reported frequently in the literature.[1,5,13]

Until recently, sex was not thought to be of prognostic value in chordomas.[5,6] O'Connell *et al.*,[14] however, suggested that female sex was an independent predictor of shortened survival in base of skull chordomas. The same observation was reported by Thieblemont *et al.*,[15] in a study of 26 cases of chordomas in various sites. They defined a favourable group of males younger than 60 years, and an unfavourable group of females and males older than 60 years. We were unable to reproduce this observation in our study. Given the small number of patients in their study, the prognostic value of combined gender and age reported by Thieblemont *et al.* still has to be confirmed by other studies. Although not generally accepted, some studies have reported a trend for patients over 40 years of age to have a worse prognosis,[14] especially with base of skull chordomas.[16] In the present study, age did not have an effect on survival.

O'Connell *et al.* have found that patients with base of skull chordomas larger than 70 ml have a significantly worse survival than those with smaller tumours.[14] Although intuitively obvious, the study[14] was the first to demonstrate that quantitative volume measurement may have prognostic significance. In our study, tumour size was not correlated with survival or progression-free survival. In contrast to the study by O'Connell *et al.* we reported chordomas in various sites. The effect of tumour size could have been concealed by the fact that large tumours were located in the sacrococcygeal region, a site amenable to a more radical surgical excision than the base of the skull. Saxton classified his patients according to tumour size (tumours < 4 cm or > 4 cm diameter) and found no difference in outcome.[17] It should be noted, however, that while computed tomography or magnetic resonance scanning were routinely used to estimate tumour size beginning in the late 1970s, various other methods were adopted to estimate tumour size from X-rays in the earlier days. Therefore, the reported median tumour sizes can only be regarded as an approximation.

**Table 1.** *The 5-year survival results of chordoma patients according to treatment modality*

| Treatment | Number of patients | Overall survival (%) | Progression-free survival (%) | Symptom-free survival (%) |
|---|---|---|---|---|
| Radiation | 9 | 41 | 34 | 30 |
| Surgery | 11 | 33 | 30 | 17 |
| Combined | 16 | 50 | 33 | 27 |

**Table 2.** *The results of a univariate analysis (log-rank test) on the influence of various variables on survival, progression-free survival and symptom-free survival in patients with chordomas*

| Variable | Overall survival | Progression-free survival | Symptom-free survival |
|---|---|---|---|
| Age (60 years) | NS | NS | NS |
| Tumour size (7 cm) | NS | NS | NS |
| Tumour site | 0.0017 | NS | NS |
| Sex | NS | NS | NS |
| Surgical margin | NS | NS | NS |
| Dose (75 Gy BED) | NS | NS | NS |
| Treatment modality | NS | NS | NS |

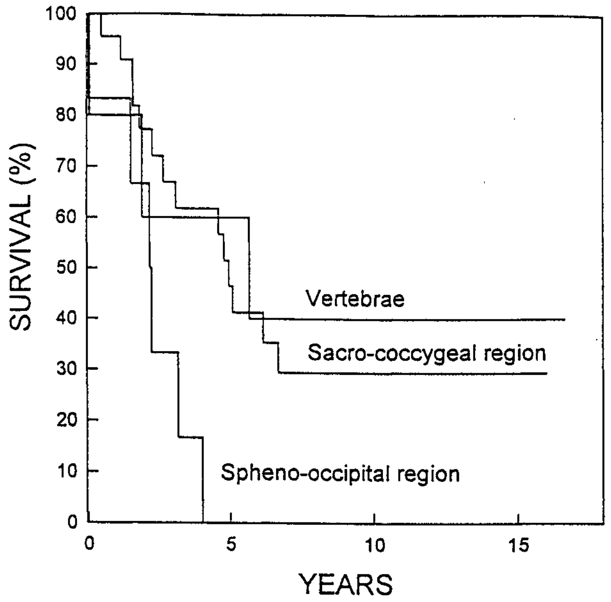

**Fig. 2.** *Actuarial (Kaplan–Meier) estimate of overall survival for chordoma patients according to site of tumor.*

Pathological features such as ploidy were reported to show a statistical trend for prognostic significance in chordoma patients.[3] Also, chondroid chordoma subtype is known to carry a better prognosis.[16] None of the cases reported here was described as chondroid chordoma. The metastasis rate of 23% reported here confirms the potential metastatic nature of chordomas stated in most of the recent literature.[1,4–6]

The factors that can affect radiation response such as total dose, radiation quality and technique varied significantly in this small cohort of patients. This may explain the absence of any correlation between the radiation parameters and any of the studied endpoints. To compensate for the variation in fractionation schedules the total dose was calculated in terms of BED. The $\alpha/\beta$ ratio of 7 Gy for chordoma was chosen arbitrarily. The same value was used previously by Tai *et al.* who also failed to demonstrate a dose–response relationship.[18] Changing the $\alpha/\beta$ ratio value did not change the overall results.

Treatment of recurrence had a life-prolonging effect as seen by the longer median survival in the treated groups. Treatment options were limited, however, by practical problems such as operability and the initial radiation dose.

The survival and progression-free survival figures reported here are similar to those reported in the literature.[1,4–6] Despite advances in surgical techniques and the technology of radiotherapy, these figures have not changed much in the last 25 years. It is important to emphasize that even after a long survival time, late recurrences can occur and few of the patients alive would be without active disease. More disturbing is the fact that even fewer patients are actually symptom free.

None of our patients with spheno-occipital chordomas survived for 5 years. This could be explained in part by the absence of chondroid chondromas in this series, the favourable histological subtype in this material. These poor survival results illustrate the limited ability of conventional treatments for chordomas of the base of the skull. A recent study using charged heavy particle beam radiotherapy reported a 5-year local control rate of 58%.[19]

We conclude that effective treatments for chordomas are still lacking. Although a group of patients may be long-term survivors. very few are symptom-free and in general the prognosis is rather poor. A multi-institutional prospective registration study may provide more information on the optimal work-up and treatment of this rare disease.

### Acknowledgements

The study was supported by the Clinical Research Unit, Danish Cancer Society, Oncologic Center, Aarhus University Hospital, Denmark.

### References

1 Eriksson B, Gunterberg B, Kindblom LG. Chordoma: a clinicopathologic and prognostic study of a Swedish National series. *Acta Orthop Scand* 1981; 52:49–58.

2 Magrini SM, Papi MG, Marletta, F. *et al.* Chordoma—natural history, treatment and prognosis. *Acta Oncol* 1992; 31:847–51.

3 Schoedel KE, Matinez AJ, Mahoney TM, *et al.* Chordomas: pathological features; ploidy and silver nucleolar organizing region analysis. A study of 36 cases. *Acta Neuropathol* 1995; 89:139–43.

4 Cummings BJ, Essen S, Harwood AR. The treatment of chordomas. *Cancer Treat Rev* 1982; 9:299–311.

5 Perez CA, Amendola BE, Lindberg R, *et al.* Unusual nonepithelial tumours of the head and neck. In: Perez CA, Brady LW, eds. *Principles and practice of radiation oncology*. Philadelphia: J.B. Lippincott, 1992:762–89.

6 Hirschfeld A, Kornblith P. Uncommon tumours of the nervous system. In: Williams CJ, Krikorian JG, Green MR, *et al.* eds. *Textbook of uncommon cancer*. Chichester: John Willey & Sons, 1988:529–630.

7 Bjornsson J, Wold LE, Ebersold MJ, *et al.* Chordoma of the mobile spine. *Cancer* 1993; 71:735–40.

8 Jacob HE. Chemotherapy for cranial base tumours. *J Neur Oncol* 1994; 20:327–35.

9 Kaplan E, Meier P. Non parametric estimation for incomplete observations. *J Am Statist Assoc* 1958; 53:457–81.

10 Cox DR. Regression models and life tables. *J R Stat Soc* 1972; 34:187–220.

11 Bethke KP, Neifeld JP, Lawrence JRW. Diagnosis and management of sacroccyoeal chordoma. *J Surg Oncol* 1991; 48:232–8.

12 Cummings BJ, Hodson DI, Bush RS. Chordoma: the results of megavoltage radiation therapy. *Int J Radiat Oncol Biol Phys* 1983; 9:633–42.

13 Keisch ME, Garcia DM, Shibuya RB. Retrospective long-term follow-up analysis in 21 patients with chordomas of various sites treated at a single institution. *J Neurosurg* 1991; 75:374–7.

14 O'Connell JX, Renard LG, Liebsch NJ, *et al.* Base of skull chordoma: a correlative study of histologic and clinical features of 62 cases. *Cancer* 1994; 74:2261–7.

15 Thieblemont C, Biron P, Rocher F, *et al.* Prognostic factors in chordoma: role of postoperative radiotherapy. *Eur J Cancer* 1995; 31:2255–9.

16 Mitchell A, Schiethauer BW, Krishnan Unni K, *et al.* Chordoma and chondroid neoplasms of the spheno-occiput. *Cancer* 1993; 72:2943–9.

17 Saxton JP. Chordoma. *Int J Radiat Oncol Biol Phys* 1981; 7:913–15.

18 Tai PTH, Caighead P, Badgon F. Optimization of radiotherapy for patients with cranial chordoma. *Cancer* 1995; 75:749–56.

19 Castro JR, Linstadt DE, Bahary JP, *et al.* Experience in charged particle irradiation of tumours of the skull base: 1977–1992. *Int J Radiat Oncol Biol Phys* 1994; 29:647–55.
